# A Pressure and Temperature Dual-Parameter Sensor Based on a Composite Material for Electronic Wearable Devices

**DOI:** 10.3390/mi14030690

**Published:** 2023-03-21

**Authors:** Zhidong Zhang, Huinan Zhang, Qingchao Zhang, Xiaolong Zhao, Bo Li, Junbin Zang, Xuefeng Zhao, Tiansheng Zhang

**Affiliations:** 1 Key Laboratory of Instrumentation Science & Dynamic Measurement of Ministry of Education, North University of China, Taiyuan 030051, China; zdzhang@nuc.edu.cn (Z.Z.);; 2 School of Software, North University of China, Taiyuan 030051, China; 3 State Key Laboratory of ASIC and System, Shanghai Institute of Intelligent Electronics & Systems, School of Microelectronics, Fudan University, Shanghai 200433, China; 4 Shanxi Hospital of Acupuncture and Moxibustion, Taiyuan 030006, China; zts910@163.com

**Keywords:** dual-parameter flexible sensor, PEDOT:PSS, CNTs, external stimuli response, wearable devices

## Abstract

Wearable sensors integrating multiple functionalities are highly desirable in artificial wearable devices, which are of great significance in the field of biomedical research and for human–computer interactions. However, it is still a great challenge to simultaneously perceive multiple external stimuli such as pressure and temperature with one single sensor. Combining the piezoresistive effect with the negative temperature coefficient of resistance, in this paper, we report on a pressure–temperature dual-parameter sensor composed of a polydimethylsiloxane film, carbon nanotube sponge, and poly(3,4-ethylenedioxythiophene)-poly(styrenesulfonate). The proposed multifunctional sensor can stably monitor pressure signals with a high sensitivity of 16 kPa^−1^, has a range of up to 2.5 kPa, and also has a fast response time. Meanwhile, the sensor can also respond to temperature changes with an ultrahigh sensitivity rate of 0.84% °C^−1^ in the range of 20 °C to 80 °C. To validate the applicability of our sensor in practical environments, we conducted real-scene tests, which revealed its capability for monitoring = human motion signals while simultaneously sensing external temperature stimuli, reflecting its great application prospects for electronic wearable devices.

## 1. Introduction

With the ever-increasing demand for electronic wearable devices, the development of multifunctional tactile sensors has shown significant progress [1,2,3,4]. Likewise, many scientific researchers have reported on flexible tactile sensors applied in human motion monitoring, pulse detection, and temperature stimuli perception. The goal of these multifunctional tactile sensors is to accurately identify different external stimuli, and then respond to them with high sensitivity and in a timely manner. Over the years, various well-performing single-function pressure sensors have been reported [4,5,6,7,8,9,10], which commonly employ piezoresistive materials whose working mechanism depends on variations in the geometric structure, tunneling resistance, or contact resistance, such as carbon nanotubes [11], carbon black nanoparticles [12], and MXene [13,14]. Nevertheless, as the key for efficient and compact electronic wearable devices, the realization of a multifunctional tactile sensor requires the integration of different types of materials with different physical properties, thereby enabling the ability to simultaneously respond to various stimuli such as pressure and temperature [15,16].

Carbon nanotube sponge, as a high-strain material with a hollow architecture, is an excellent choice for the fabrication of pressure sensors. The typical structural hierarchy enables the CNT sponge to keep its shape, without requiring the support of substrates or electrodes [11,12,17]. Moreover, the microstructure of the CNT sponge consists of CNTs with a large number of micropores uniformly distributed within. This unique internal distribution results in low density and thermal conductivity. Furthermore, poly(3,4-ethylenedioxythiophene)-poly(styrenesulfonate) is an excellent temperature-sensitive material [18,19,20,21,22,23]. Its advantages are its high conductivity and strong stability, making it a common material for fabricating flexible temperature sensors. However, previous studies have focused on the application of PEDOT:PSS for improved conductivity, while ignoring its own temperature coefficient of resistance. Additionally, by adding certain organic solvents or an organic polyhydroxy, such as DMSO (dimethyl sulfoxide), ethylene glycol, or glycerol, the electrical conductivity of a polymer can be increased dozens or even hundreds of times over. Additionally, the high adhesion strength of PEDOT:PSS allows it to easily form a film on the surface of the CNT sponge.

In this work, we fabricated a multifunctional sensor by first drying the CNT sponge soaked in PEDOT:PSS in the oven. By doing so, the PEDOT:PSS formed a film on the surface of the CNT sponge without destroying its internal structure. The PEDOT:PSS-coated CNT sponge was then sandwiched between two polydimethylsiloxane films to complete the fabrication process. The fabricated pressure–temperature dual-parameter sensor had high sensitivity rates of 16 kPa^−1^ in the low-pressure range and 2.5 kPa^−1^ in the range of 2 kPa to 10 kPa. Even applied to ultrahigh pressure (40 kPa), our sensor showed a sensitivity rate of 0.32 kPa^−1^. Within a temperature range of 20 °C to 80 °C, the sensor had an ultrahigh sensitivity rate of 0.84% °C^−1^.

## 2. Experimental Section

### 2.1. Materials

The PDMS (Sylgard 184 silicone elastomer base and related curing agents) was purchased from Dow Corning Co., Ltd., Midland, MI, USA. The CNT sponge was purchased from Nanjing XFNANO Materials Tech Co., Ltd., China. The PEDOT:PSS was purchased from Shanghai Aladdin Bio-Chem Tech Co., Ltd., China. All materials were used without any further purification.

Parameters:

CNT sponge: Inner diameter: 10–20 nm; outer diameter: 30–50 nm; porosity: 99%;

PEDOT:PSS: Viscosity: 6.0000 mPa.S; solid content: 1.40000%; PH(20 °C): 1.9900.

### 2.2. Fabrication of PDMS Flexible Substrate

To obtain a flexible PDMS substrate with a thickness of about 1mm, the main and curing agents of the PDMS were mixed at a mass ratio of 10:1 in a plastic cup. Next, the compound reagent was refrigerated for 1 h to remove any bubbles in it. A four-inch silicon wafer was then cleaned with alcohol and dried in the oven. Afterwards, the PDMS mixture was spin-coated onto the silicon wafer at a speed of 300 r/min for 120 s. Then, the wafer was placed on a hot plate at 100 °C for 1 h, where the PDMS flexible substrate was left uncovered. After cooling, the PDMS film was peeled off and used as the flexible substrate.

### 2.3. Fabrication of CNT Sponge Coated with PEDOT:PSS Film

A 0.5 cm × 0.5 cm CNT sponge was cut and soaked in the PEDOT:PSS for 1 h. The CNT sponge was placed on a clean wafer and dried in the oven for half an hour at 80 °C, after which it was cooled down to complete the fabrication.

### 2.4. Preparation of the Sensor

One 3 cm × 1 cm PDMS film was cut as the bottom substrate and another 0.5 cm × 0.5 cm film was cut as the top substrate. One 1.5 cm × 0.8 cm electrode was used to receive the current signals. The CNT sponge coated with PEDOT:PSS was laid flat over the interdigital electrode to ensure a close contact, and then the whole structure was sandwiched by two PDMS films. Then, a conductive silver paste with high conductivity was used to connect the pins of interdigital electrodes with the wires for measurements. After drying the silver paste for 1 h at 80 °C in the oven, the preparation of the multifunctional sensor was completed.

### 2.5. Characterizations and Measurements

The surface and cross-sectional images of the PDMS film, CNT sponge, and CNT sponge wrapped by PEDOT:PSS were taken using field emission scanning electron microscopy (SEM SUPRA-55 purchased from Shanghai Opton Co., Ltd., China). Moreover, the qualitative analysis of the surface element distribution of the CNT sponge coated with PEDOT:PSS was performed via energy-dispersive spectrometry (EDS). Meanwhile, a Kethley 2450 controlled digital source meter was used to measure the real-time I-T and I–V curves.

## 3. Results and Discussions

### 3.1. Fabrication and Characterization

**Figure 1:**
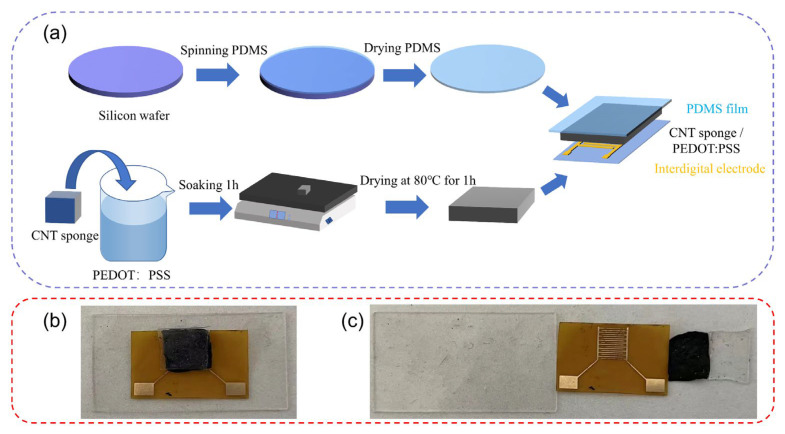
(**a**) Fabrication process of the pressure–temperature dual-parameter sensor. (**b**) Real image of the sensor. (**c**) Real image of each part of the sensor.

As shown in Figure 1a, the complete fabrication process can be mainly divided into two parts. One part involves the preparation of PDMS film, which is a common substrate for flexible sensors. The PDMS mixture was spin-coated onto a silicon wafer uniformly, and after its dried, the PDMS film was peeled off and used as the flexible substrate. The preparation of the composite sponge was relatively simple. After soaking the CNT sponge in PEDOT:PSS solution, a thin film was formed on its surface. The sponge was then dried in the oven while keeping it uncovered. The PEDOT:PSS-coated sponge was sandwiched by two PDMS films, and the composite CNT sponge was closely attached to the interdigital electrode for better conductivity. As shown in Figure 1b,c, the sensor consists of four parts, which from bottom to top are the PDMS film, interdigital electrode, composite CNT sponge, and PDMS film.

**Figure 2:**
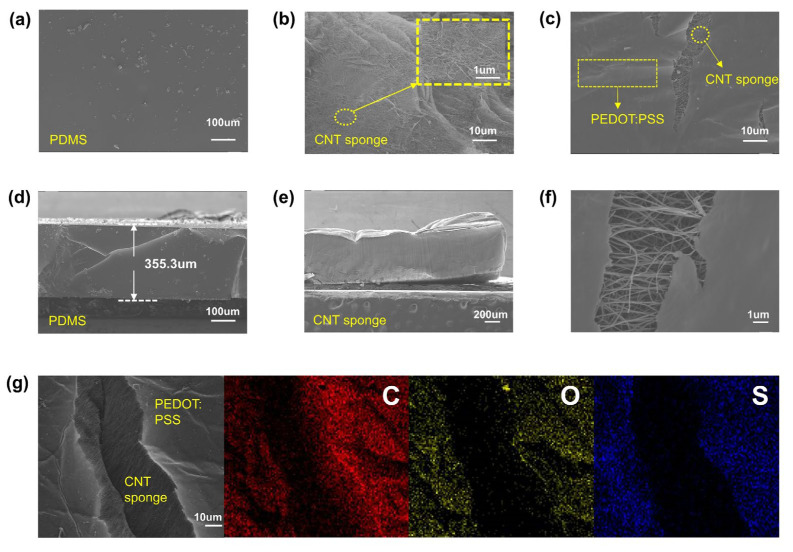
SEM images of the materials used in this work: (**a**) SEM image of the surface of the PDMS substrate; (**b**) SEM image of the surface of the CNT sponge; (**c**) SEM image of the surface of the CNT sponge wrapped in PEDOT:PSS; (**d**) measured thickness of the PDMS substrate; (**e**) measured thickness of the CNT sponge; (**f**) SEM image of the surface of the CNT sponge wrapped in PEDOT:PSS at higher resolution; (**g**) energy-dispersive spectroscopy (EDS) mapping images of the CNT sponge wrapped in PEDOT:PSS.

PDMS is a commonly used material for the preparation of flexible sensors due to its high dielectric constant and tunable elasticity [17,24,25,26,27,28,29,30]. Additionally, its stress–strain relationship can be adjusted by varying the mixing ratio of the main PDMS agent and curing agent in the fabrication. As shown in Figure 2a,d, the cured PDMS film has a flat surface and a thickness of 355.3 um.

The CNT sponge is a self-assembled and interconnected conductive material with a sponge-like shape. Due to its light weight and excellent flexibility, they are commonly used as the dielectric layer in the sensor. As shown in Figure 2b, the CNT sponge consists of many carbon nanotubes and has a porosity of up to 99%. Despite the excellent thermal conductivity of single carbon nanotubes, the low density and high thermal resistance at the CNT junctions mean CNT sponge has extremely low thermal conductivity, nearly unaffected by temperature in pressure sensing [27]. Moreover, Figure 2e indicates that the CNT sponge used in this experiment has a thickness of about 1 mm. Progressively, PEDOT:PSS has received a great deal of attention owing to its unique properties, including its transparency, relatively high conductivity, and simple fabrication process. Over recent years, many research studies have focused on its thermoelectric properties and characteristics, such as its electrodes, while its thermistor-type behavior has been less explored [26]. Herein, we fabricate a composite sponge by soaking the CNT sponge in PEDOT:PSS. It can be seen from Figure 2c,f that the PEDOT:PSS is coated on the CNT sponge as an ultrathin film. Notably, very few PEDOT:PSS molecules get into the CNT sponge and most are distributed on the sponge surface. To verify this, we took an EDS mapping image (Figure 2g), where it can be seen that the C and O elements are distributed throughout the crack in the CNT sponge coated with PEDOT:PSS, but there is almost no S element (according to the molecular structure of PEDOT:PSS, the S element is unique compared to the CNT sponge).

The IDE structure is used because of its own good flexibility, bending resistance, and conformal ability. Using the interdigital electrode as the signal response channel of the sensor, the flexible substrate, MXene film, and interdigital electrode can be closely adhered to accurately measure the pressure signal generated by the human body or other carriers. Moreover, the interdigital electrode has the characteristics of multiple conductive channels, so that the breakage or warping of an electrode will not affect the overall output performance of the sensor, thereby greatly improving the stability and reliability of the sensor.

### 3.2. Pressure Sensing Performance of CNT Sponge

**Figure 3:**
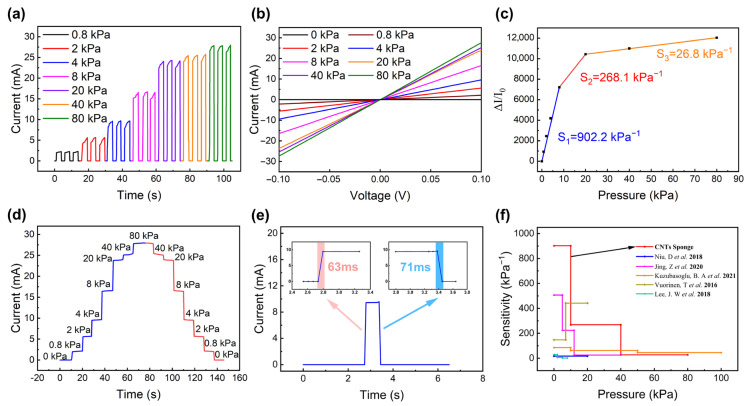
The pressure sensing properties of the CNT sponge: (**a**) the I-T curves at pressures less than 80 kPa; (**b**) the linear relationship of the I–V curves at voltages from −0.1 V to 0.1 V; (**c**) the sensitivity of the CNT sponge, reaching 902.2 kPa^−1^ below 10 kPa, 268.1 kPa^−1^ in the transitional pressure region (10 kPa to 20 kPa), and 26.8 kPa^−1^ in the high pressure region (20 kPa to 80 kPa); (**d**) gradient of the rise and fall of the current in the pressure region from 0 kPa to 80 kPa; (**e**) the response time of the CNT sponge for loading and unloading pressure; (**f**) the performance comparison with other pressure-sensitive materials [21,22,23,24,25].

The CNT sponge is a widely used piezoresistive material that has excellent conductivity. To verify its piezoresistive properties, the interdigital electrodes are used to receive the electrical signals and a digital SourceMeter is used to monitor the electrical responses under different pressure values. As shown in Figure 3a, the I-T curves at pressures less than 80 kPa indicate that the current increases with the pressure, reflecting a negative piezoresistive nature. When the deformation occurs, the internal structure of the CNT sponge is compressed, resulting in smaller pores and the exclusion of air. The carbon nanotubes approach each other under pressure and the resistance becomes smaller. The linear relationship between the current and voltage shown in Figure 3b indicate that an ohmic contact forms between the interdigital electrode and the CNT sponge. However, when the applied pressure becomes higher, the amplitude of the current increase starts to decrease. Moreover, as shown in Figure 3c, the CNT sponge exhibits different sensitivity levels in different pressure regions. This is because at low pressure, the amount of contact in the cross-linked network structure inside the sponge is less and the whole structure is relatively loose. When a small amount on pressure is applied to the sponge, the microstructures in the internal cross-linked network structure start to contact each other. Hence, the conductive path expands rapidly, and the resistance change rate increases greatly. In contrast, when the applied pressure is further increased to a larger value, the growth rate of the resistance change becomes smaller, since the cross-linked network structure inside the conductive sponge has already been fully contacted and the conductive path has stabilized. When continuous pressure is applied at an interval of 10 s, the CNT sponge shows good gradient changes, as shown in Figure 3d. Additionally, the CNT sponge has a fast response time of 63 ms, while the recovery time is 71 ms, as shown in Figure 3e. Recently, different piezoresistive materials have been reported, and compared to them [21,22,23,24,25], the sensor from this work can better monitor the pressure and temperature, simultaneously, with high sensitivity.

The pressure sensitivity is determined by the variation of the current and applied pressure. The specific calculation formula is as follows:

(1) $$S=(\frac{I_{p}-I_{0}}{I_{0}})/P$$

where, *S* (kPa^−1^) is the sensitivity; *I*_0_ (mA) is the measured current without pressure; *I_p_* (mA) is the measured current when pressure is applied; *P* (kPa) is the value of applied pressure.

### 3.3. Temperature-Sensing Performance of CNT Sponge Wrapped by PEDOT:PSS

**Figure 4:**
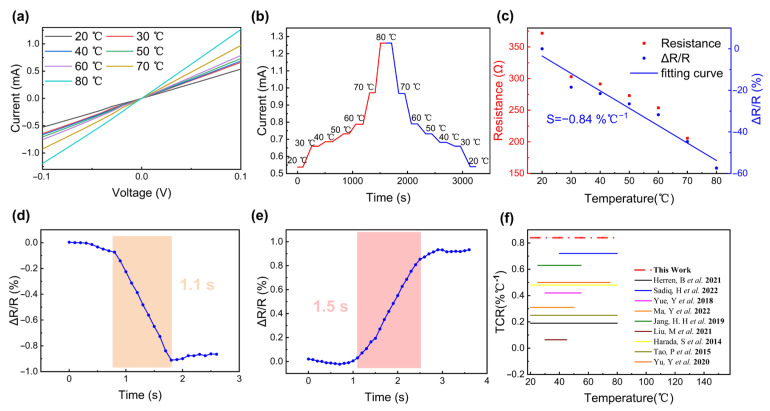
The temperature−sensing properties of the CNT sponge wrapped in PEDOT:PSS: (**a**) the linear relationship of the I–V curves at voltages from −0.1 V to 0.1 V; (**b**) the gradient of the current increase and decrease when under a continuous temperature change; (**c**) the resistance and resistance change rate of the device over a temperature range from 20 °C to 80 °C, exhibiting a sensitivity of 0.84% °C^−1^; (**d**) the response time of the composite sponge when the temperature rises; (**e**) the response time of the composite sponge when temperature drops; (**f**) comparison with other studies [13,14,17,20,26,29,30,31,32].

There have been two types of carbon-based temperature sensors reported recently: positive temperature coefficient (PTC) and negative temperature coefficient (NTC) sensors. PEDOT:PSS is a typical organic semiconductor material with NTC-type temperature-sensitive characteristics, whose sensing mechanism is based on the semiconductor properties of the material. When the ambient temperature is low, the number of carriers in the NTC-sensitive material is relatively small, resulting in higher resistance. However, as the temperature increases, the heat energy increases, thereby activating more carriers and reducing the resistance. To verify this property, we carried out a series of tests. As shown in Figure 4a, the linear relationship of the I–V curves indicates that the PEDOT:PSS film has a close contact with the interdigital electrode. Meanwhile, the rising trend of the current with increasing temperature indicates a negative temperature coefficient of resistance. Additionally, when the temperature rises, the current shows an upward gradient trend, as shown in Figure 4b. Furthermore, as evident from Figure 4c, in the temperature range of 20 °C to 80 °C, the resistance drops by about 210 ohms and the resistance change is about 60%, yielding a sensitivity of 0.84% °C^−1^. In addition, as the temperature rises by about 1 °C, the sensor responds in about 1.1 s (Figure 4d). However, when the temperature drops by 1 ℃, the response time of the sensor is larger, around 1.5 s (Figure 4e). In recent years, various thermo-sensitive materials have been reported, such as PEDOT:PSS/CuPc, PEDOT:PSS/IPA, and PEDOT:PSS/PDMS. Compared to these reported materials [13,14,17,20,26,29,30,31,32], the material used in our work offers a wider temperature range and a higher sensitivity level, as shown in Figure 4f.

The temperature sensitivity is determined by the variation of resistance and the applied pressure. The specific calculation formula is as follows:

(2) $$S=\left(\frac{R_{T}-R_{0}}{R_{0}}\right)/\Delta T\;$$

where, *S* (% °C^−1^) is the sensitivity; *R*_0_ (Ω) is the initial resistance under 20 °C; *R_T_* (Ω) is the tested resistance when temperature changes; *ΔT* (°C) is the value of the temperature variation.

### 3.4. Sensing Performance of the Sensor When Pressure and Temperature Are Applied Simultaneously

**Figure 5:**
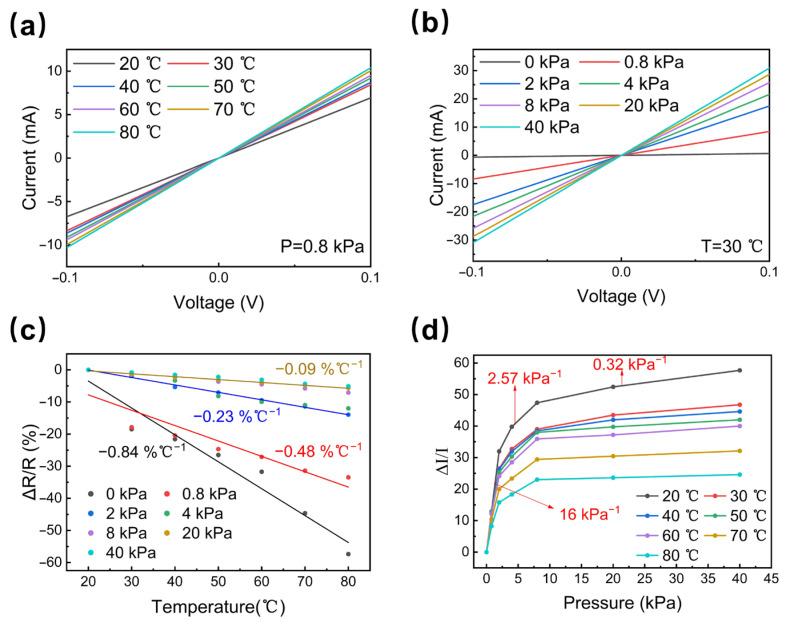
Performance of pressure–temperature co-detection: (**a**) I–V curves in the temperature range of 20–80 °C at 0.8 kPa; (**b**) I–V curves in the pressure range of 0 kPa to 40 kPa at 30 °C; (**c**) resistance change rates and temperature sensitivity curves under different pressures in the range of 0 kPa to 40 kPa; (**d**) current change rates and pressure sensitivity curves at different temperature values in the range of 20–80 °C.

To verify the capability of the device to simultaneously handle both pressure and temperature stimuli, we used a digital source meter to detect the I-T curves in a temperature range of 20 °C to 80 °C at 0.8 kPa, and also in a pressure range of 0 kPa to 40 kPa at 30 °C. As shown in Figure 5a, the overall current change shows an increasing trend with the temperature, and the increase rate is small. However, a larger increase rate can be observed with the pressure, as shown in Figure 5b. This is because of the difference in thermal resistance and piezoresistive mechanisms [33], which have different degrees of influence on the resistance. Likewise, the same trend is also reflected in the sensitivity curves. As indicated in Figure 5c,d, the pressure and temperature sensitivity curves both tend to decrease, where the temperature sensitivity curves drop with a faster rate. The reason behind this could be as follows. As shown in Figure 3b and Figure 4a, the device deformation led by pressure has a greater effect on the change in current than the temperature. Thus, when testing the temperature sensitivity curves under a fixed pressure, the current change induced by the pressure is larger, thereby resulting in a stronger decreasing trend. Meanwhile, when testing the pressure sensitivity curves at a fixed temperature, the current change caused by the temperature is smaller, thereby resulting in a weaker decreasing trend. In general, the device has excellent pressure and temperature sensitivity, and is suitable for multifunctional monitoring. Compared with the previous work, the sensor we prepared performs well in the pressure-sensing range, temperature-sensing range, and pressure response time (shown in Table 1). In the next experiment, we plan to complete the decoupling of the pressure and temperature.

**Figure 6:**
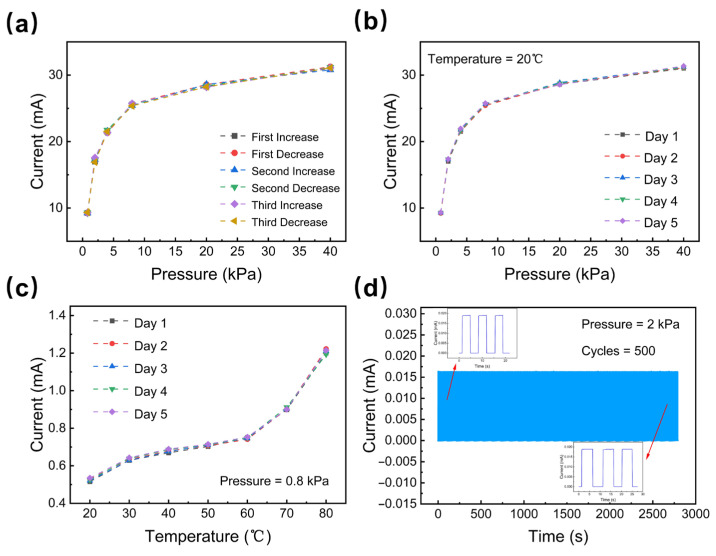
The durability and stability of the sensor (**a**). The pressure hysteresis of the sensor (**b**) and (**c**) the stability of the sensor. (**d**) The durability of the sensor.

Nowadays, wearable electronic devices have broad application prospects in many fields, the key to which is to meet the requirements for the durability and stability of the sensor. We carried out a series of experiments to verify this. The three consecutive pressure increases and decreases prove that the sensor has a small hysteresis range, as shown in Figure 6a. As shown in Figure 6b,c, we tested the pressure and temperature-sensing performance of the sensor for five consecutive days. The results exhibit good stability. Additionally, with the help of the pressing machine, we performed 500 cycles of repeated experiments of pressure loading and release, the results of which are shown in Figure 6d.

### 3.5. Practical Applications of the Multifunctional Sensor

**Figure 7:**
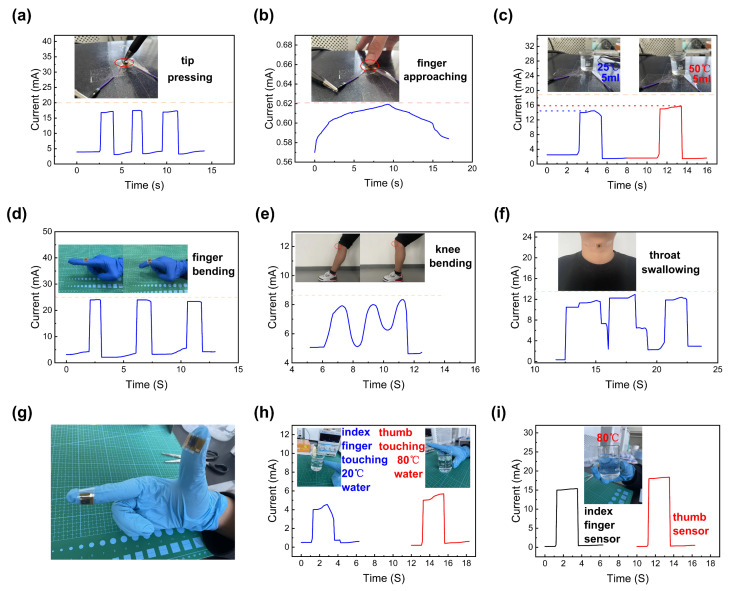
Real-scene tests of the sensor: (**a**) I-T curve when pressing the sensor with a pen tip; (**b**) I-T curve when finger approaches the sensor; (**c**) I-T curve when placing the sensor on a 5 mL glass of 25 °C water and 5 mL of 50 °C water; (**d**) I-T curve when the finger bends; (**e**) I-T curve when the knee bends; (**f**) I-T curve while swallowing; (**g**) the wearable sensors; (**h**) I-T curves of the two sensors when touching the water at different temperatures; (**i**) I-T curves of the two sensors when holding a beaker with 80 °C water.

Ideally, a pressure–temperature multifunctional sensor should have the ability to monitor these two external stimuli simultaneously [38,39]. To verify this property, we conducted experiments involving external contact to the sensor and human motion monitoring. As shown in Figure 7a, we applied a large pressure to the sensor three times through the tip of a pen, and the sensor responded to the stimulus precisely and stably in all three attempts. In Figure 7b, we demonstrate the temperature detection capability of the sensor in response to an approaching finger. It should be noted that because of the PDMS film’s thermal insulation, it took about 10 s for the sensor to respond. In order to analyze the pressure–temperature co-interaction of the sensor, we chose two glasses of water of the same mass but at different temperatures for the experiment. As shown in Figure 7c, the increase in temperature resulted in an obvious increase in the current. Meanwhile, some experiments related to human motion monitoring were also carried out. As shown in Figure 7d–f, the I-T curves corresponding to finger bending, knee bending, and throat swallowing experiments all exhibit a continuous and stable signal. Essentially, good monitoring performance for pressure and temperature is the key to achieving efficient wearable devices. As shown in Figure 7g,h, we attached the sensors to the fingers and measured the current of the two sensors when touching the water at different temperatures. The larger current induced when touching the water with higher temperatures indicated that the sensor can precisely respond to a change in temperature. Additionally, when holding the beaker with 80 °C water, the thumb sensor showed a larger current than the rest because of the difference in pressure, which in turn reflects the sensitivity of the sensor to pressure change (shown in Figure 7i). As evident from these results, this multifunctional sensor based on composite materials has great application prospects in wearable devices.

## 4. Conclusions

In this work, we reported on a pressure–temperature multifunctional sensor with high sensitivity and a fast response time. The sensor is mainly composed of CNT sponge wrapped by PEDOT:PSS and interdigital electrodes, and sandwiched between two ultrathin PDMS films. In real-scene tests, this sensor showed excellent performance, i.e., a high sensitivity level of 16 kPa^−1^ in the low-pressure region and an ultrahigh sensitivity rate of 0.84% °C^−1^ in the temperature range of 20 °C to 80 °C. Meanwhile, it also exhibited a fast response time to both pressure (63 ms) and temperature (1.1 s) stimuli compared to other studies. Additionally, we also conducted experiments with external stimuli and involving human motion monitoring to verify the applicability of this sensor for use in wearable devices. Owing to the good piezoresistive and thermoresistance effects of the materials, coupled with our unique fabrication scheme, this multifunctional sensor can play a pivotal role in future wearable devices.

## Figures and Tables

**Table 1:** A comparison with the previous work [34,35,36,37].

Materials	Working Range (kPa)	Response Time (ms)	Temperature Range (K)	Independent of P and T Signals	Ref.
PVDF/PANI/PDMS	10	–	0–100	Yes	1
Silk fiber-AgNW & CNTs/ionic liquid	2	250	30–65	Yes	2
Ionnic hydrogel	1	500	30–55	No	3
Metal-organic porous carbon/PDMS	2	60	20–100	No	4
**CNTs sponge/PECOT:PSS/PDMS**	**40**	**63**	**20–80**	**No**	**This work**

## Data Availability

The data that support the findings of this study are available upon reasonable request from the authors.
